# Insights into ALD Growth of Al-Based Dielectric Stack on 4H-SiC

**DOI:** 10.3390/nano16120782

**Published:** 2026-06-22

**Authors:** Bruno Galizia, Emanuela Schilirò, Patrick Fiorenza, Filippo Giannazzo, Bela Pecz, Zsolt Fogarassy, Fabrizio Roccaforte, Raffaella Lo Nigro

**Affiliations:** 1 Consiglio Nazionale delle Ricerche—Istituto per la Microelettronica e Microsistemi (CNR-IMM), Strada VIII 5, Zona Industriale, 95121 Catania, Italy; bruno.galizia@imm.cnr.it (B.G.); emanuela.schiliro@imm.cnr.it (E.S.); patrick.fiorenza@imm.cnr.it (P.F.); filippo.giannazzo@imm.cnr.it (F.G.); fabrizio.roccaforte@imm.cnr.it (F.R.); 2 Centre for Energy Research, Institute of Technical Physics and Materials Science, Konkoly-Thege ut 29-33, 1121 Budapest, Hungary; pecz.bela@ek.hun-ren.hu (B.P.); fogarassy.zsolt@ek.hun-ren.hu (Z.F.)

**Keywords:** atomic layer deposition, aluminum nitride, aluminum oxide, in situ ellipsometry

## Abstract

An Al_2_O_3_/AlN stack deposited via Atomic Layer Deposition (ALD) methods as a gate insulator for silicon carbide (4H-SiC) has been investigated, focusing on the effects of different Al_2_O_3_ deposition processes on the nitride layer. In particular, dielectric stacks, consisting of a 10 nm AlN interface (001)-oriented layer directly grown on a 4H–SiC substrate and in 20 nm of additional amorphous Al_2_O_3_ layers were synthesized in sequential deposition runs by thermal ALD (T-ALD) or plasma-enhanced ALD (PEALD) methods. The evolution of the phenomena occurring at the Al_2_O_3_/AlN interfaces has been established by in situ ellipsometry measurements. Strong effects of the oxygen plasma because of the O-Al-N bond formation have been clearly observed and corroborated by ex situ structural and electrical characterizations, especially in the case of the plasma-enhanced Al_2_O_3_ process. In particular, the Al_2_O_3_/AlN bilayer grown by the Al_2_O_3_ T-ALD method exhibited good insulating behavior and an 8.7-high dielectric constant was measured. By contrast, the Al_2_O_3_/AlN bilayer grown by the Al_2_O_3_ PEALD method demonstrated poor insulating properties.

## 1. Introduction

In recent decades, insulating materials with high dielectric permittivity values (high-κ) have attracted an increasing interest both in the academic and industrial communities due to their promising properties for applications in a wide range of fields such as in metal oxide thin film transistors [1], in flexible electronics [2], in OLED displays [3], and in solar cells as passivation layers [4], as well as in energy storage systems [5].

Regarding microelectronics applications, the scaling of metal oxide semiconductor field effect transistors (MOSFETs), predicted by Moore’s law, has been, for many decades, the driving force for the integration of high-κ dielectrics in silicon technology [6,7]. However, modern power electronics applications are now moving towards wide band gap semiconductors, such as silicon carbide or gallium nitride, where the use of high-κ insulators is also desired to improve the transistors’ performances in terms of on-resistance, breakdown voltage and device transconductance [8].

In particular, silicon carbide (4H-SiC) is an ideal candidate for replacing silicon in high-voltage MOSFETs [9], but its high breakdown field is not fully exploitable due to the low dielectric constant value of the commonly used silicon oxide (SiO_2_) gate dielectric [10,11]. In this context, aluminum oxide (Al_2_O_3_) and aluminum nitride (AlN) can be interesting alternatives to SiO_2_ to decrease the device’s derating [10].

Al_2_O_3_ shows high dielectric constant values (~7–10) [8,12], a large critical electric field (~9–10 MV/cm) [13] and good thermal stability and amorphous structure [14]. However, the quality of the Al_2_O_3_/SiC interface is typically worse than the SiO_2_/SiC system. In fact, many studies demonstrated the need for a thin SiO_2_ interlayer between the Al_2_O_3_ insulator layer and SiC semiconductor to improve the gate dielectric performance [8,12]. On the other hand, AlN also showed a high dielectric constant (~9) [15] and good band offset [16]. In addition, since it possesses small in-plane lattice mismatch (~0.9%) and a nearly identical thermal expansion coefficient with respect to 4H-SiC, it could potentially guarantee high interface quality [17,18].

In our previous work, we reported on the growth of different highly (0001) oriented AlN thin layers on (0001)4H-SiC and their electrical behavior [19]. However, the presence of AlN grain boundaries could act as current pathways, favoring leakage current and consequent early device breakdown [20,21,22,23].

In this context, the Al_2_O_3_/AlN dielectric stack could result in improved interface quality because of the (0001)AlN preferential orientation on 4H-SiC and of the Al_2_O_3_ amorphous layer as a blocking layer for leakage current. Moreover, the Al_2_O_3_/AlN is a “fully high-k” stack, thus avoiding the SiO_2_ limitation in the perspective of high-voltage applications [24]. However, AlN exhibits a significant affinity towards oxygen, leading to the formation of a non-stoichiometric aluminum oxynitride (AlO_x_N_1−x_), both on its surface and throughout the bulk [25,26]. Interestingly, it has been shown that different oxygen content can have an influence on both the oxynitride’s structural and electrical properties [27,28,29]. For instance, Takeuchi et al. [30] demonstrated the importance of controlling the oxygen content in the AlO_x_N_1−x_ dielectric layer by tuning the composition via different ALD processes and correlating it with different dielectric properties of the final MIS capacitors. Therefore, concerns should be directed to possible induced oxidation of AlN layer during Al_2_O_3_ deposition and its effect on the Al_2_O_3_/AlN stack’s dielectric properties.

To date, ex situ techniques were typically reported for chemical characterizations of AlN deposited layers, but alternative in situ characterizations would provide more accurate control and contaminant-free measurements.

This present paper is devoted to the chemical and electrical properties of Al_2_O_3_/AlN stack on 4H-SiC, focusing on the effect of ALD Al_2_O_3_ growth by different methods (i.e., thermal as well as plasma-enhanced processes) on the oxidation of the thin AlN interfacial layer deposited on silicon carbide. In particular, non-destructive in situ ellipsometry has been used “in operando” mode for the real-time characterization of both dielectric materials deposited via ALD [31]. Specifically, in situ ellipsometry was used to monitor thickness growth and variation of the refractive index during AlN and Al_2_O_3_ layer depositions. Then, the in situ investigation was correlated to the results obtained by complementary ex situ characterization techniques, namely scanning transmission electron microscopy (STEM) coupled with energy dispersion spectroscopy (EDS). Finally, the dielectric properties of the two Al_2_O_3_/AlN/4H-SiC capacitors were compared and correlated to the effects of different oxygen exposure during deposition process.

## 2. Materials and Methods

In this experiment, commercially available n-type 4° off-axis (0001)-oriented 4H-SiC epitaxial layers (doping concentration ND = 8 ×10^15^ cm^−3^, thickness t = 6.7 μm) grown onto a heavily doped 4H–SiC substrate were used. Before each ALD deposition, every 2 cm × 2 cm 4H-SiC coupon was cleaned with piranha solution (H_2_SO_4_:H_2_O_2_ = 1:3) for 10 min and diluted hydrofluoric acid (H_2_O:HF = 10:1) for 5 min to remove eventual carbon contaminations and residual native oxide. ALD processes for dielectric stack deposition were carried out on a PE-ALD LL SENTECH Instruments GmbH reactor equipped with a remote capacitively coupled plasma (CCP) source and excited by a 13.56 MHz RF generator via a matchbox with a power supply of 200 W.

Process parameters are summarized in Table 1. Firstly, a thin (~10 nm) AlN layer was deposited at 300 °C using trimethylaluminum (TMA) precursor as Al source and NH_3_ plasma as reacting gas for nitrogen. A 40 sccm N_2_ flow was used as carrier and as purging gas and each cycle was repeated 115 times. After the deposition of the AlN layer, the deposition temperature was decreased to 250 °C without vacuum breaking, so that the sequentially ~20 nm Al_2_O_3_ thin film was deposited on the AlN layer by the 250 cycle T-ALD or by 180 cycle PE-ALD processes.

Phenomena occurring at the Al_2_O_3_/AlN interface during the early stage of Al_2_O_3_ depositions were monitored by introducing in situ ellipsometric measurements at end of every ALD cycle. For this purpose, a Sentech SE 801 in situ spectroscopic ellipsometer operating (Berlin, Germany) in UV-VIS wavelength range (between 200 and 1040 nm) at 70° single angle of incidence was used to acquire psi (Ψ) and delta (Δ) spectra. SENTECH Spectraray4 software was used for data modelling and regression analysis, in which fit quality was monitored as deviation between measured and modelled spectra as mean squared error (MSE).

The dielectric behavior of the interface and stack was also evaluated by electrical measurements on metal insulator semiconductor (MIS) capacitors. The MIS capacitors, having an active area of 7.854 ×10^−5^ cm^2^, were fabricated using 80 nm thick sputtered Ni/Au electrodes, defined via optical photolithography and lift-off. Capacitance–voltage (C-V) and current–voltage (I-V) measurements were carried out at 1 kHz, because of low series resistance contribution, using a Microtech Cascade probe station equipped with a Keysight B1505 parameter analyzer.

Finally, both the Al_2_O_3_/AlN bilayers deposited on (0001) 4H-SiC were investigated as cross-sectioned samples by focused ion beam (FIB) (SCIOS2 SEM + FIB dual beam manufactured by ThermoFisher, Brno, Czech Republic) using scanning transmission electron microscopy (STEM) and energy dispersion spectroscopy (EDS) performed with an aberration-corrected Titan Themis 200 microscope (ThermoFisher, Eindhoven, The Netherlands).

## 3. Results and Discussion

Two different Al_2_O_3_/AlN dielectric stacks were deposited on (0001)4H-SiC epitaxial layers by sequential ALD processes of the Al compounds. In particular, the interfacial AlN (~10 nm) layers were always deposited via PE-ALD process, while the following Al_2_O_3_ (~20 nm) layers have been grown using either T-ALD (sample A) or PE-ALD (sample B).

**Figure 1:**
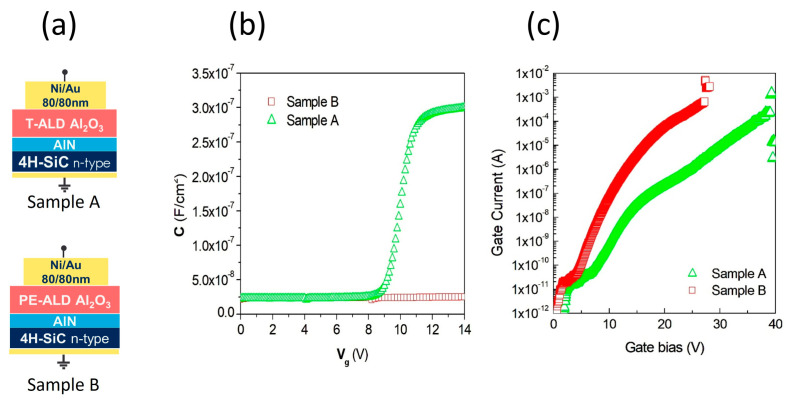
Schematical images of fabricated MIS (**a**). C–V curves (**b**) and I-V measurements (**c**) acquired for sample A (green triangles) and sample B (red squares).

The electrical behavior of both Al_2_O_3_/AlN/4H-SiC samples A and B has been investigated by fabrication of MIS capacitors (Figure 1a) using Ni/Au metal gate. Capacitance–voltage (C-V) curves (Figure 1b) have been acquired at 1 kHz to compare the electrical quality of the two stacked samples. Interestingly, while sample A showed a typical C-V curve shape reaching the accumulation capacitance in the positive gate bias range, measurements in sample B resulted in a very flat C-V curve, never reaching the accumulation condition. The complete and quantitative analysis of the dielectric properties of sample A indicated a dielectric constant value of 8.7, which represents a quite good and high value. Nevertheless, the C-V curve showed a significant positive shift of the flat band voltage (VFB = +7.55 V). This shift can be due to a negative fixed charge (N_eff_), which has been estimated to be N_eff_ = 1.4 × 10^13^ cm^−2^, or, alternatively, it could be attributed to the presence of slow near-mid-gap acceptor states at the SiC interface as demonstrated in our previous study on p- and n-type MOS capacitors [24].

On the other hand, Figure 1c shows current/voltage measurements (I/V) performed using the same MIS structures fabricated on samples A and B. The two I/V curves seem to be almost overlapped in the initial 0–7.5 V range, but, at voltages higher than 7.5 V, a different conductivity is quite evident. The thicknesses of samples A and B are adequate in order to camper the current magnitudes [32] and it is possible to affirm that sample B shows both higher current values an earlier breakdown with respect to sample A. This further investigation can be considered the explanation for the missed accumulation in the C/V characterization curve of sample B.

**Figure 2:**
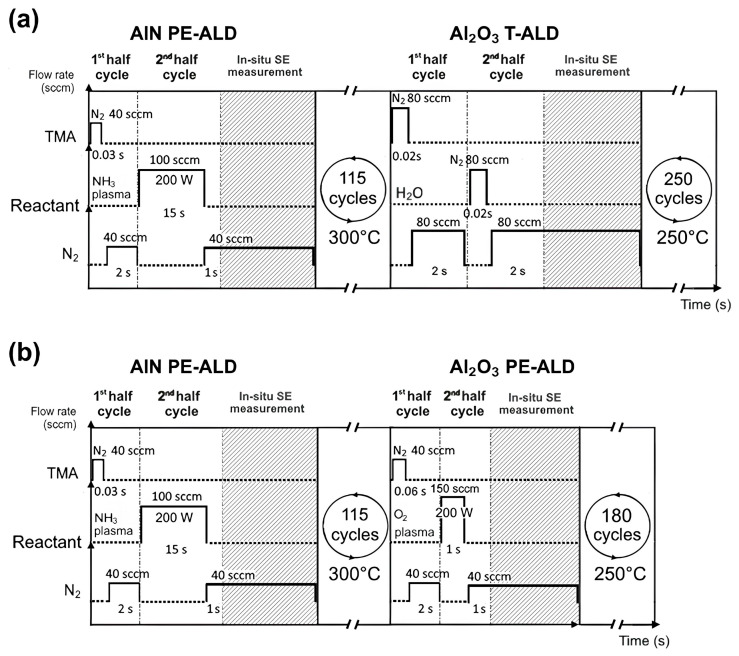
ALD cycle deposition parameters and in situ cyclic SE measurements for (**a**) plasma-enhanced ALD of AlN and thermal-ALD Al_2_O_3_ (sample A) as well as for (**b**) plasma-enhanced ALD of both AlN and Al_2_O_3_ (sample B).

However, since the reason for the electrical behavior of sample B is not clear, the origin of the degradation of its MIS electrical performances deserves to be investigated. In this context, attention has been paid to the phenomena occurring at the Al_2_O_3_/AlN interfaces during the deposition processes. In fact, it is possible that oxygen ions might diffuse within the AlN layer by O^2−^ occupation of N-vacancies [33] and O-Al-N bonds are likely formed; thus, insights on the phenomena occurring at the Al_2_O_3_/AlN interface during the deposition processes have been collected for both samples A and B. The possible formation of an AlO_x_N_1−x_ layer has been investigated by in situ SE at the end of every ALD cycle (Figure 2) on small-size silicon substrates, which were inserted in the deposition chamber alongside the 4H-SiC substrate. Real-time measurements were performed during the whole deposition process of the AlN and Al_2_O_3_ layers as well as on the last 80 cycles of the AlN deposition and on the sequentially initial 80 cycles of the Al_2_O_3_ layers.

**Figure 3:**
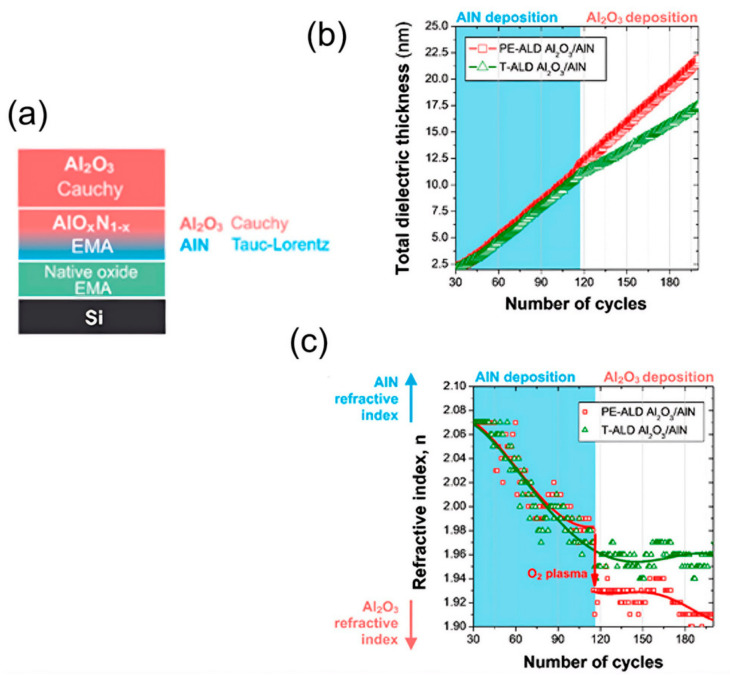
(**a**) Schematic illustrations of the optical models used for SE data fitting and (**b**) film thickness values vs. the number of ALD cycles for sample A (green triangles) and sample B (red squares). (**c**) Evolution of the refractive index at 633 nm with the number of ALD cycles for samples A and B. The shaded blue rectangular area represents the AlN PE-ALD process, whereas Al_2_O_3_ depositions are reported with white background.

Different dispersion models (Figure 3a) are widely used for each deposited layer, namely the Cauchy layer model for the transparent Al_2_O_3_ [34,35] and the Tauc-Lorentz dispersion for the AlN, which has a slight adsorption in the short wavelength range [36,37]. The possible formation of an AlO_x_N_1−x_ layer has been modelled via an effective medium approximation optical model (EMA), which calculates the final optical properties as a combination of different materials averaging the refractive indexes (n_EMA_) of the two Al_2_O_3_ and AlN materials using Equation (1), where (f) represents the volume fractions of inclusions [38].

(1) $${n_{EMA}}^{2}=\frac{\sum_{i=1}^{N}f_{i}\cdot {n_{i}}^{2}}{\sum_{i=1}^{N}f_{i}}$$

In particular, during nitride deposition, fitting parameters were EMA layer thickness and volume fractions of inclusions, whereas, during oxide deposition, the Al_2_O_3_ thickness and EMA volume fractions of inclusions were fitted. Firstly, film thickness values upon increasing the number of ALD cycles have been evaluated and reported in Figure 3b. A constant growth-per-cycle (GPC) trend, which is the typical feature of the ALD regime growth, has been observed for all the processes and the extracted values, obtained as slopes of the thickness-cycle straight lines, which are: GPC = 1.1 Å/cycle for AlN by PE-ALD process, while GPC = 1.2 Å/cycle and GPC = 0.8 Å/cycle values have been calculated for Al_2_O_3_ by PE-ALD and T-ALD, respectively. These values are in good agreement with previous data reported in the literature [39].

Hence, the calculated AlO_x_N_1−x_ refractive index values as a function of ALD number of cycles is reported in Figure 3c and, for all the fitted data, mean squared error (MSE) remained below 0.95, indicating good reliability. During nitride deposition (blue background), refractive index values equally stay constant in both samples for about 50 cycles, and then it decreases as Al_2_O_3_ deposition starts. In particular, for sample B (red squares), the nitride deposition end corresponds to a drastic reduction in refractive index value that further decreases during oxide deposition. On the other hand, during the initial cycles of Al_2_O_3_ thermal deposition for sample A, the refractive index (green triangles) smoothly decreases as well but reaches a plateau at higher values. This difference could be explained by considering pure Al_2_O_3_ and AlN refractive index values.

Indeed, aluminum nitride has a refractive index at 633 nm of 1.9–2.1 in its polycrystalline phase, which is consistently higher than that of amorphous Al_2_O_3_ (1.6–1.7 for as-deposited ~20 nm layer). All in situ data described above pointed to a clear interaction at the Al_2_O_3_/AlN interface, especially in the case of the deposition process for sample B.

**Figure 4:**
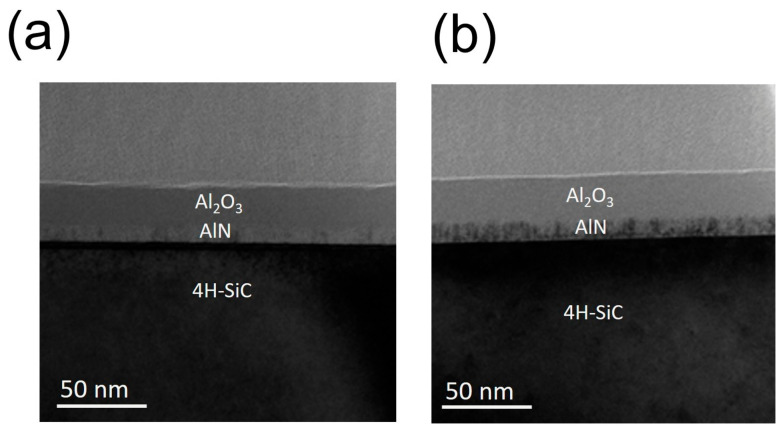
STEM images of the two different dielectric stacks deposited on 4H-SiC: (**a**) sample A and (**b**) sample B.

Hence, structural film properties and the quality of their interfaces have been imaged by scanning transmission electron microscopy (STEM). Low magnification images of samples A (Figure 4a) and B (Figure 4b) showed uniform coating; the crystalline nature of AlN is clearly visible, whereas Al_2_O_3_ layers appear amorphous in both samples. The AlN layers are about 10 nm thick, while the Al_2_O_3_ layers are 18–19 nm thick for samples A and B in both the oriented (001) AlN planes, showing a perfect alignment with respect to those of the (0001)4H-SiC substrate as previously demonstrated [19]. On the other hand, some differences are evident at the Al_2_O_3_/AlN interface, which is quite sharp in sample A and almost porous in sample B.

**Figure 5:**
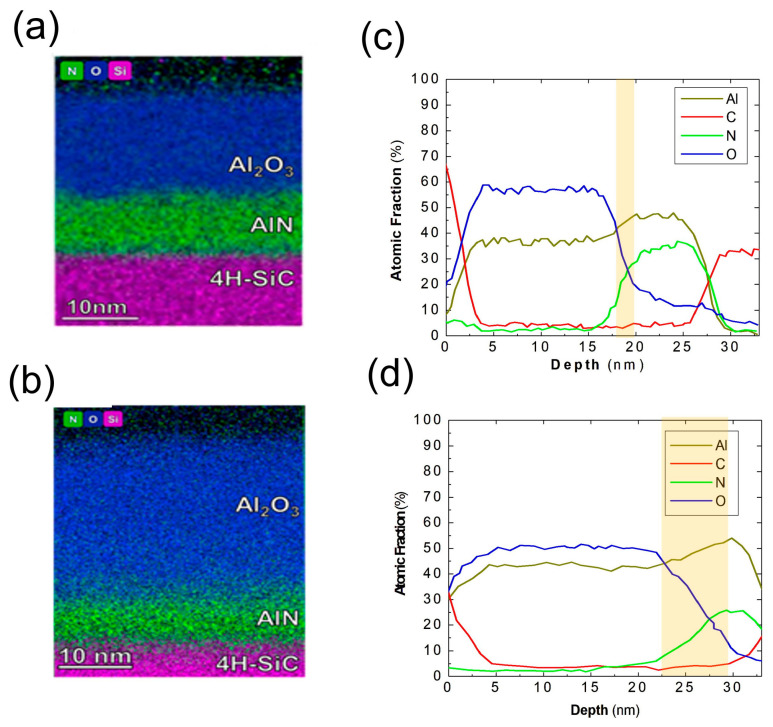
STEM-EDS maps showing the elemental distribution of N, O, and Si across the layers are provided in (**a**) for sample A and (**b**) for sample B and the relative depth profiles of elemental distribution of Al, C, N, and O, obtained in (**c**) sample A (T-ALD Al_2_O_3_) and (**d**) sample B (PE-ALD Al_2_O_3_). The shaded yellow regions represent the overlapping of the O and N signals, indicating the presence of non-stoichiometric AlO_x_N_1−x_ layers at the interfaces.

This difference was investigated by EDS measurements performed on cross-sectional samples. Chemical maps are reported in Figure 5a and Figure 5b for samples A and B, respectively. The principal difference between samples A and B consists in the nitrogen and oxygen distribution at the Al_2_O_3_/AlN interfaces. As a matter of fact, the nitrogen map of sample B appears to be slightly thinner (~7–8 nm) than in sample A (9–10 nm), while the oxygen map clearly demonstrates the occurrence of an oxygen diffusion from the Al_2_O_3_/AlN layer into the underlying AlN layer in the case of the PE-ALD process. Oxygen radicals were easily diffused and an oxidation process probably occurred at the Al_2_O_3_/AlN interface in sample B.

The distribution of the chemical elements inside the two Al_2_O_3_/AlN stacks has also been observed by the spatially resolved STEM-EDS signals elaborated as chemical depth profiles. The trends of carbon, aluminum, nitrogen and oxygen atomic fraction percentages from film surface (0 nm) to the underlying SiC (35 nm) have been monitored and are reported in Figure 5c,d. The presence of carbon at the film surface is due to the organic glue used during the fabrication of sample cross-sections. The overlapping of Al and O signals and their ratio clearly confirm the presence of the Al_2_O_3_ layers. However, different oxide layer thicknesses produced by the T-ALD and PE-ALD processes have been again observed and, more interestingly, while the oxygen signal decreases and the nitrogen rises at the interface with the AlN, an overlap of the N and O signals occurs. This is highlighted as yellow areas in Figure 5c and Figure 5d related to samples A and B, respectively. The composition within the yellow areas indicated the formation of a non-stoichiometric oxynitride layer such as AlO_x_N_1−x_ and the thickness of this “transition layer” is very different between the A and B samples. Therefore, these profiles demonstrated that the oxygen contamination occurred at a much deeper thickness in sample B with respect to sample A.

**Figure 6:**
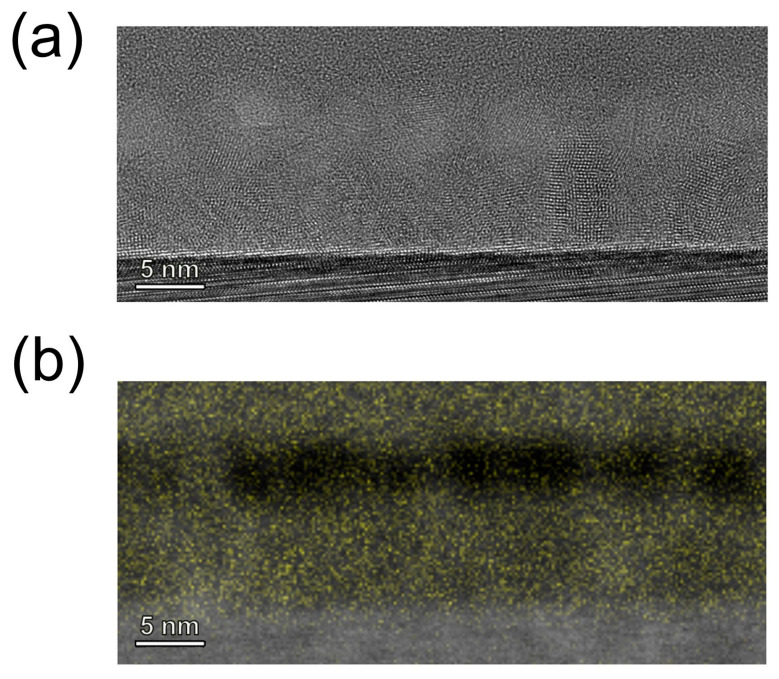
STEM image (**a**) of sample B and (**b**) the relative EDS aluminum map.

Furthermore, in Figure 6, STEM image and the relative aluminum map by STEM-EDS analysis have also been acquired at the Al_2_O_3_/AlN interface for sample B. In particular, it is characterized by visible irregularities already observed at a lower magnification in Figure 4b, but, here, they are clearly observable from the lack of the Al signal in Figure 6b representing the Al elemental map acquired at 490 keV.

In this context, Yeh and Tuan [40] demonstrated the formation of micropores by the surface oxidation of AlN by oxygen, producing gaseous nitrogen and Al_2_O_3_/AlN with a reaction that is more thermodynamically favored than water-induced AlN oxidation. The presence of oxygen in the plasma phase during deposition probably followed a similar reaction pathway, energetically favoring the reaction even more.

The persistence of the diffraction fringes indicates that the crystalline phase of the AlN film is preserved after Al_2_O_3_ deposition, indicating that the AlN layer retains its initial crystallinity throughout the process. The images suggest that the interaction with the oxygen plasma likely induces a modification of the interfacial density, which is consistent with the pronounced change in refractive index measured by in situ ellipsometry. This interpretation is further supported by the microstructural observations, which reveal an apparent reduction in the initial AlN thickness while maintaining its crystalline nature, together with the formation of an interfacial region where the aluminum distribution appears less homogeneous. In this context, we can affirm that the fringes are not related to the Al_2_O_3_ layer, but to the AlN; in fact, they are present within the initial AlN film thickness.

It is clear that both STEM and the related chemical analysis provides a comprehensive investigation into the structural and chemical properties of the deposited materials; but, they represent ex situ and destructive characterization methods, while the oxygen contamination in the aluminum nitride layer has been also detected by the in situ ellipsometry measurements.

Finally, all these collected data by both chemical and electrical characterization demonstrated that the total high-k Al_2_O_3_/AlN stack is an interesting and promising system with respect to the parental Al_2_O_3_ layer.

## 4. Conclusions

In summary, Al_2_O_3_/AlN stacks deposited by ALD on 4H-SiC have been investigated, focusing on the effects of different Al_2_O_3_ deposition processes on the AlN layer. In particular, in situ spectroscopic ellipsometry was demonstrated as a valuable non-destructive technique for the real-time monitoring of the oxygen contamination in AlN thin films during the T-ALD and PE-ALD deposition of an overlying Al_2_O_3_ thin layer. By modelling the AlN optical properties as an oxynitride material (AlO_x_N_1−x_), the oxygen content was progressively correlated with the measured real-time varying oxynitride refractive index values. These findings were consistent with complementary ex situ STEM-EDS analysis carried out on samples processed from the same ALD conditions on 4H-SiC, validating the reliability of in situ ellipsometry. The significance of the proposed approach was further corroborated by the electrical characterization of MIS capacitors made from the deposited dielectric stack samples. Indeed, C-V and I-V measurements showed that strong oxidation at the AlO_x_N_1−x_ surface can cause critical degradation of the dielectric properties. While a quantitative correlation remains to be established in future studies, these results pave the way for the use of in situ ellipsometry in setting the processing conditions for high-k dielectrics in power electronics device processing.

## Figures and Tables

**Table 1:** Deposition parameters for the depositions of the AlN and Al_2_O_3_ layers.

Stack	Layer	ALD Method	TMA Pulsing Time (s)/Purging Time (s)	NH_3_ Plasma or H_2_O, or O_2_ Plasma Pulsing Time (s)/Purging Time (s)	Number of Cycles
Sample A	AlN	PE ALD	0.03/2	15/1	115
	Al_2_O_3_	Thermal-ALD	0.02/2	0.02/2	250
Sample B	AlN	PE ALD	0.03/2	15/1	115
	Al_2_O_3_	PE-ALD	0.06/2	1/1	180

## Data Availability

The data are available from the corresponding author upon reasonable request.
